# Functional near-infrared spectroscopy for the assessment of overt reading

**DOI:** 10.1002/brb3.100

**Published:** 2012-10-28

**Authors:** Dima Safi, Maryse Lassonde, Dang Khoa Nguyen, Phetsamone Vannasing, Julie Tremblay, Olivia Florea, Olivier Morin-Moncet, Mélanie Lefrançois, Renée Béland

**Affiliations:** 1 École d'orthophonie et d'audiologie, Université de MontréalMontréal, Canada; 2 Centre de recherche en neuropsychologie et cognition, Université de MontréalMontréal, Canada; 3 Centre de recherche de l'Hôpital Sainte-Justine, Hôpital Sainte-JustineMontréal, Canada; 4 Service de neurologie, Hôpital Notre-Dame du CHUMMontréal, Canada

**Keywords:** Adults, irregular words, lexical reading, nonwords, optical imaging, phonological reading, reading aloud

## Abstract

Functional near-infrared spectroscopy (fNIRS) has become increasingly established as a promising technique for monitoring functional brain activity. To our knowledge, no study has yet used fNIRS to investigate overt reading of irregular words and nonwords with a full coverage of the cerebral regions involved in reading processes. The aim of our study was to design and validate a protocol using fNIRS for the assessment of overt reading. Twelve healthy French-speaking adults underwent one session of fNIRS recording while performing an overt reading of 13 blocks of irregular words and nonwords. Reading blocks were separated by baseline periods during which participants were instructed to fixate a cross. Sources (*n* = 55) and detectors (*n* = 16) were placed bilaterally over frontal, temporal, parietal, and occipital regions. Two wavelengths were used: 690 nm, more sensitive to deoxyhemoglobin (HbR) concentration changes, and 830 nm, more sensitive to oxyhemoglobin (HbO) concentration changes. For all participants, total hemoglobin (HbT) concentrations (HbO + HbR) were significantly higher than baseline for both irregular word and nonword reading in the inferior frontal gyri, the middle and superior temporal gyri, and the occipital cortices bilaterally. In the temporal gyri, although the difference was not significant, [HbT] values were higher in the left hemisphere. In the bilateral inferior frontal gyri, higher [HbT] values were found in nonword than in irregular word reading. This activation could be related to the grapheme-to-phoneme conversion characterizing the phonological pathway of reading. Our findings confirm that fNIRS is an appropriate technique to assess the neural correlates of overt reading.

## Introduction

Functional near*-*infrared spectroscopy (fNIRS) has become an increasingly promising imaging technique for mapping cortical activation related to cognitive tasks. This technique allows the measurement of hemodynamic responses associated with neuronal activity by projecting near-infrared light at two different wavelengths (between the 650- and 900-nm spectrum), then recording intensity modulations of the reflected light from each wavelength that are absorbed by oxygenated (HbO) and deoxygenated (HbR) hemoglobin (Villringer and Chance 1997; Gratton et al. 2000). It has been used in various research domains with pediatric and adult populations without any neurological disorders (Watson et al. 2004; Gallagher et al. 2007; Kovelman et al. 2008) as well as with epileptic participants (Watanabe et al. 1998; Gallagher et al. 2007, 2008; Ota et al. 2011; see Dieler et al. 2012 for a review).

The fNIRS studies conducted with healthy adults have mainly focused on the hemodynamic changes associated with language-related processes. Hull et al. (2009) examined cortical activity in bilateral temporal regions during an overt picture-naming task in 10 English-speaking healthy adults. fNIRS recordings were not affected by verbalization artifacts and the results revealed robust activation in the left temporal region with no significant changes in the analogous right-hemisphere region. Ehlis et al. (2007) used a verbal fluency task (letter and category) to investigate changes in the concentrations of HbO and HbR in the left hemisphere (including prefrontal, temporal, and central regions) in a group of 12 healthy participants. The participants exhibited strong increases of [HbO] in large areas of the left frontal cortex while performing the overt verbal fluency task during three 30-sec periods. Gallagher et al. (2007) also used the overt verbal fluency task to determine language lateralization in two healthy adults (one right-handed and one left-handed). For both participants, the results showed strong initial activation in Broca and Wernicke areas during the language task, followed by weaker activation in the corresponding areas of the right hemisphere, suggesting a left lateralization of language. In Chaudhary et al. (2011)'s study, 15 healthy right-handed adults also performed an overt verbal fluency task. The results revealed an increase in [HbO] and a decrease in [HbR] in the anterior frontal cortex with more activation in the left than right anterior frontal cortex, whereas activation was bilateral in the prefrontal cortex. The verbal fluency task also demonstrated consistent results in functional magnetic resonance imaging (fMRI) and magneto-encephalography (MEG) in determining language localization and lateralization (Yamamoto et al. 2006; Pelletier et al. 2011; Gallagher et al. 2008; Pirmoradi et al. 2010).

With regard to reading, a recent review by Quaresima et al. (2012) reports on fNIRS studies that used overt or covert reading in adults. For example, Kahlaoui et al. (2007) presented 112 written words (concrete nouns) and 112 written pseudowords to 10 adults aged between 25 and 35. While undergoing fNIRS recordings, the participants were asked to read the stimuli silently and decide whether each stimulus was a word that belonged to the French language. fNIRS data showed increased blood oxygenation patterns in frontal and temporal regions bilaterally (increase of [HbO] and decrease of [HbR]) in the decision phase. Another study by Hofmann et al. (2008) examined cortical oxygenation changes in the superior frontal gyrus (SFG), the left inferior parietal gyrus (IPG), and the left inferior frontal gyrus (IFG) while German-speaking participants were performing a lexical decision task from visual input. The stimuli set consisted of 100 written words (50 low-frequency and 50 high-frequency) and 100 written pseudowords. The results showed a significantly larger [HbO] increase and [HbR] decrease in the SFG and left IPG in word than in pseudoword reading. In the IFG, a significantly greater decrease of [HbR] without an increase of [HbO] was found when participants read low-frequency words, compared with high-frequency words. The author's hypothesis is that the decrease of [HbR] is likely due to the contribution of the grapheme-to-phoneme conversion that is higher when reading low- versus high-frequency words. In addition to words and pseudowords, sentence and text stimuli were also used in fNIRS studies. For instance, Kennan et al. (2002) conducted NIRS recordings while six healthy adult participants were requested to make a grammatical judgment on written sentences. Half of the sentence stimuli were well-formed while the other half were either syntactically or semantically incongruous. The results revealed a left-hemisphere language dominance in prefrontal areas. In Horovitz and Gore (2004)'s study, participants silently read a technical manual during fNIRS recording that showed activation in Broca and Wernicke areas in five of the six participants. The Liu et al. (2008)'s study is one of the few fNIRS studies in which participants were tested in overt reading. The researchers asked 22 healthy participants to read an unfamiliar text out loud for 5 min. fNIRS recordings in the bilateral prefrontal regions revealed an hyperoxygenation, defined as [HbO] levels three standard deviations higher than those at rest, in 15 of the 22 participants, and hypooxygenation, defined as three standard deviations lower than the level measured at rest, in seven participants. In Lo et al. (2009)'s study, participants read aloud continuously for 2 min a 50-word passage from a medical journal. A significant increase of [HbO] compared with the baseline was recorded in the left motor cortex without changes in [HbR].

The functional neuroanatomy of word and nonword reading has been examined using fMRI. As fMRI is highly sensitive to movement and verbalization artifacts, the majority of studies used silent reading tasks (Mechelli et al. 2000; Chen et al. 2002; Heim et al. 2005). For instance, in a study by Joubert et al. (2004), 10 healthy French-speaking participants underwent one session of fMRI recording while reading silently. Activation related to silent reading of nonwords, high-frequency, and low-frequency words was distributed within a network of posterior temporoparietal, inferior frontal, and middle and superior temporal regions bilaterally. In addition, nonwords and low-frequency words elicited a significantly higher activation in bilateral inferior frontal gyri than high-frequency words. In the fMRI study by Mechelli et al. (2005), English-speaking participants silently read regular words, irregular words, and pseudowords. The reading of the pseudowords tended to increase the activation in the left dorsal premotor area, whereas the reading of the irregular words tended to increase the activation in the left pars triangularis. In comparison with the reading of regular words, activation in the left pars opercularis was higher when participants read both irregular words and pseudowords. There are a few fMRI studies using overt reading tasks where researchers adapted the acquisition data procedure to minimize artifacts due to head motion during overt speech. For instance, in Dietz et al. (2005)'s study, participants read aloud when MRI gradients were turned off to minimize movement artifacts during image acquisition. Covert and overt reading of English regular words (monosyllabic nouns of mid-range frequency) and pseudowords induced a significant activation relative to baseline (fixation of a cross) in the left precentral gyrus and the left ventral occipitotemporal region. In both the left IFG and the left intraparietal sulcus, a higher level of activation was found for pseudowords than for words. When comparing overt and covert reading, overt reading elicited larger increases in the BOLD response than covert reading in the premotor, motor, auditory, and extrastriate cortex bilaterally. In another fMRI study (Borowsky et al. 2006), the presentation of the written exception words and pseudohomophones was followed by a periodic 1650-msec gap in image acquisition during which the participants produced the stimuli out loud, and bilateral activation for both stimuli types in the middle and inferior frontal gyri, superior temporal gyri, and occipitotemporal gyri was recorded. Seghier et al. (2008) shortened the block duration and asked their participants to whisper the responses using minimal mouth movements. While reading aloud familiar words, some participants showed activation in the left inferior frontal and anterior occipitotemporal regions while others in the right inferior parietal and left posterior occipitotemporal regions.

In summary, fMRI studies investigating reading processes, either covert or overt, revealed a large neural network that included parts of the frontal, temporal, parietal, and occipital regions bilaterally with some differences in activation when comparing word and nonword reading. On one hand, the IFG and temporoparietal areas (including the angular gyrus, supramarginal gyrus, and auditory associative cortex) appeared mostly involved in nonword reading associated with grapheme-to-phoneme conversion and phonological processing, while the occipitotemporal areas (including the inferior occipital cortex and the fusiform gyrus) seemed to be more implicated in irregular word reading associated with lexical processing.

As fMRI is highly sensitive to verbalization artifacts, it is not conducive to investigating overt reading contrary to fNIRS which is resistant to verbalization artifacts. An important advantage of overt over covert reading is that the reader's performance measured in terms of accuracy and reading speed can be accounted for. This study investigated the applicability of an fNIRS protocol in studying brain areas that subserve the reading aloud of irregular words (lexical pathway of reading) and nonwords (phonological pathway of reading) in French-speaking healthy adults. In contrast to previous fNIRS studies, an extensive coverage of the cerebral regions beyond the classical frontal and/or temporal ones was used. We expected to visualize a widespread network of reading-related activations in the frontal, temporal, parietal, and occipital regions, similar to that shown in the fMRI studies, with some differences in the activation between irregular word and nonword reading.

## Material and Methods

### Participants

We recruited 15 healthy native French speakers (six males, nine females), aged 22–50 (mean age = 28.25 years old, SD = 9.69) with a mean education of 16.25 years (SD = 2.23). Participants had no history of neurological disorders or reading difficulties. Reading age level was assessed with the *Test de l'Alouette* (Lefavrais 1967) and no participants showed deficits with respect to chronological age. All participants were right-handed (the handedness of each participant was assessed by the *Edinburgh Handedness Inventory*) (Oldfield 1971). The data from three participants (three women) were withdrawn from the analyses due to technical artifacts associated with either discomfort during testing or low optical signal due to high hair density. This study was approved by the Ethics Committees of the Sainte-Justine and Notre-Dame Hospital Centers (University of Montreal). Informed consent was obtained in writing from all participants.

### Stimuli

The stimuli consisted of 390 written irregular words (e.g., *héros* [“hero”], *monsieur* [“sir”]) and 390 written nonwords (e.g., *huyan*,*tirasate*). Irregular words included at least one grapheme that has more than one sound correspondence. For instance, in *monsieur* (“sir”), the grapheme *on* has many sound correspondences: [œ] as in *monsieur*, [õ] as in *chaton* (“kitten”), and [ 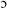] as in *canyon*] and the grapheme *os* in *héros* has at least another sound correspondence [os] as in *os* (“bone”). Nonwords were legal and pronounceable according to the spelling-to-sound correspondence rules in the French orthography. However, to avoid analogical reading, nonwords did not include any homophones and bore the least resemblance to words. Each nonword was paired with an irregular word; they were matched by length (in number of letters, graphemes, phonemes, and syllables) and syllabic structure. For instance, the irregular word *laps* [laps] was matched with the nonword *jist* [ℨist]. The 390 stimuli were distributed into 13 blocks (each block contained 30 irregular words and 30 nonwords) in a counterbalanced manner in terms of lexical frequency, phonological complexity (phonemic and syllabic structure), and length (number of letters, graphemes, and syllables). The 13 blocks are reported in Table A1 in the Appendix. Word frequency ranged from 0.07 to 1093 (New et al. 2001); number of letters, from 4 to 13; and number of syllables, from 1 to 4.

### Experimental procedure

**Figure 1:**
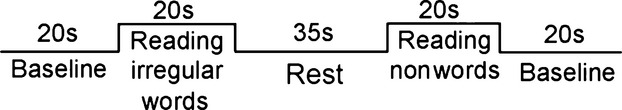
Time course for one of the 13 blocks beginning with irregular word reading.

Participants underwent one session of fNIRS recording, which took place in a dark and soundproof room. All stimuli were presented to the participants on a 20-inch computer screen using the software *Presentation* as black low-case (Arial, 40) on a light-gray background. Each participant was seated in a comfortable chair at approximately 130 cm from the computer screen. Participants were asked to read aloud and most accurately as possible the irregular words and nonwords. As soon as they produced the irregular word, the next one would appear on the screen. If an irregular word was unknown to the participant, the investigator would immediately pass to the next one in order to avoid the participants relying on the slower phonological pathway of reading. Thus, the number of stimuli presented varied according to the participant's reading speed, with a presentation time limit of 1.15 sec for irregular words and 1.5 sec for nonwords. Stimuli were presented in a block-design paradigm with each block divided as follows: a 20-sec baseline period, followed by 20 sec of reading aloud irregular words, a 35-sec resting period, 20 sec of reading aloud nonwords, and a final 20-sec resting period. As each reading block lasted 20 sec and contained a maximum of 30 irregular words or nonwords, the presentation rate did not exceed 90 irregular words or nonwords per minute. The order of the reading tasks (i.e., irregular words vs. nonwords) was counterbalanced across the 13 blocks in such a way that seven blocks began with irregular word reading and six blocks with nonword reading. The order of the blocks was also counterbalanced across participants. Figure 1 depicts the design of one block.

During the baseline and resting periods, participants were instructed to relax and minimize their thoughts while fixating a cross presented in central position on the computer screen. Individual reading speed and number of errors were recorded. Nonwords were judged as correctly produced if the participant applied the spelling-to-sound correspondences in the French language. If a nonword had more than one acceptable pronunciation (e.g., *acho* can be pronounced [aʃo] or [ako]), both pronunciations were judged as correct. The total session duration was approximately 90 min, including the setup of the helmet, sources, detectors, and electrodes.

### Data acquisition

**Figure 2:**
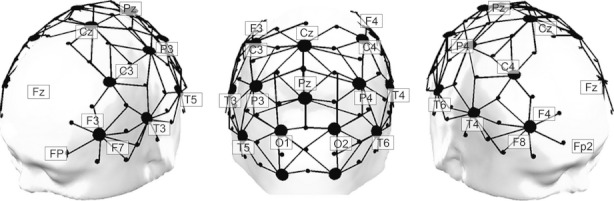
Layout of the 55 sources and 16 detectors over the left and right hemispheres used for all 12 participants. The larger circles correspond to the detectors and the smaller ones to the sources. Labels in squares represent the 10–20 international electrode placement coordinates.

Data were gathered with a rate of 19.5312 Hz using a 128-channel spectrometer *Imagent Tissue Oxymeter* (ISS Inc., Champaign, IL) that included 55 sources and 16 detectors, and the software package *Boxy* (Photon Migration Imaging Lab, Massachusetts General Hospital, MA). Two wavelengths were used: 690 nm, sensitive to HbR concentration changes, and 830 nm, sensitive to HbO concentration changes. No detector saturation occurred during the experiment. The sources and the detectors were placed on the participant's scalp using a custom-built, rigid but comfortable helmet allowing head movements and aloud verbalization. Hair around sources and detectors was parted to avoid interference with light emission and detection. Two different helmet sizes were used (57 and 59 cm) depending on the head circumference of the participants. A standard montage was created for each helmet using the software *Brainsight*^*™*^ *Frameless 39* (Rogue Research, Canada). According to the International 10–20 system (Jasper 1958), sources and detectors were placed bilaterally over cerebral regions classically involved in reading processes: Broca and Wernicke areas, the left middle and superior temporal gyri, the left parietal gyrus, the left temporo-occipital region, the left visual cortex, and their right homologous regions. The source–detector distance varied from 2 to 5 cm. Figure 2 shows the regions covered by the montage.

A simultaneous EEG recording, with four electrodes placed in accordance with the 10–20 system (Fz, Cz, Pz, and Oz), was carried out in order to control for the participants’ alert state during the task. After the recordings, the exact location of each source, detector, and EEG electrode, as well as four fiducial points (nasion, left and right preauricular, and tip of the nose), were digitized and recorded for each participant using the stereotaxic system *Brainsight* to allow individual reconstitution of the montage on a standardized MRI adult template, the Colin27 (Evans et al. 1992).

### Data analysis

fNIRS data were processed using the *HomER* (*Hemodynamic Evoked Response*) software (Huppert et al. 2009) and downsampled by a factor of 5 to lighten the data processing. The raw hemodynamic signal was normalized with a 10-sec prestimulus time. Artifact rejection took place by withdrawing segments with light intensity amplitudes smaller than 100 DC or a normalized standard deviation higher than 50%. The optical intensity of the raw data (DC) was filtered using a low-frequency zero-phase digital filtering with a high cutoff frequency at 0.1 Hz. A modified Beer–Lambert law with a differential path length factor (DPF) correction according to the age of each participant was applied (Duncan et al. 1996; Strangman 2003).

For each participant, concentration changes in HbO, HbR, and HbT were averaged across the 13 blocks. HbT was computed by summing changes in HbO and HbR. Averages were coregistered and projected on the Colin27 standard MRI template (Evans et al. 1992) to visualize the activated brain regions.

## Results

### Behavioral results

EEG monitoring revealed no signs of drowsiness for all participants while they were performing the tasks. The participants read an average of 19 irregular words (SD = 1.5) and 15 nonwords (SD = 1.4) per block for a reading speed of 57 irregular words per minute (SD = 4.5) and 45 nonwords per minute (SD = 4.2). We found that the average error rate within a block was 1.25 errors on irregular words (SD = 0.49) and 1.95 errors on nonwords (SD = 0.88). The demographic (age, gender, and years of education) and behavioral data (number of irregular words and nonwords read, number of errors produced) of the 12 participants are reported in Table 1.

**Table 1:** Demographical data (gender, age, and years of education); individual mean number of irregular words and nonwords read in the 13 twenty-second blocks and individual mean number of errors produced in reading irregular words and nonwords

Participant	Gender	Age	Years of education	Mean number of irregular words read	Mean number of errors	Mean number of nonwords read	Mean number of errors
F.M.	F	22	16	19	1.3	16	3
I.C.	F	22	16	20.5	1.3	15.3	0.9
J.N.	F	22	16	21.1	1.2	17.5	2.3
J.T.	F	26	17	17.5	1.4	13.2	2.8
C.T.	F	27	19	17.8	2.2	15.4	3.4
J.P.	F	34	16	20.5	1.4	15.5	2.5
A.C.	M	20	16	22.2	1.1	16.8	1.2
D.V.	M	23	17	20.6	2	13.6	1
N.H.	M	26	17	18.4	0.8	13.1	2.1
B.B.	M	37	20	18.5	0.5	14.8	1.8
H.C.	M	49	18	18	0.7	14.1	0.6
M.D.	M	51	16	18.9	1	14.1	1.7

### fNIRS results

#### Temporal course of the hemodynamic responses

**Figure 3:**
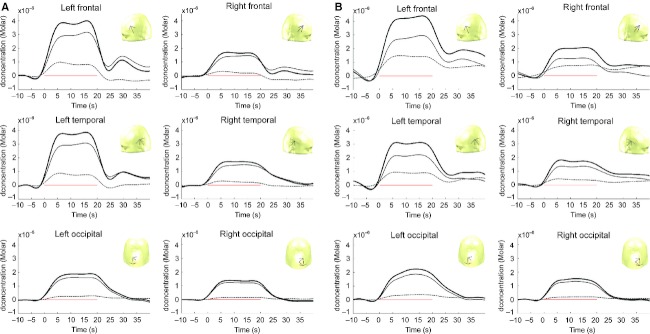
Time course (*x*-axis) of the hemodynamic responses in reading aloud for irregular words (A) and nonwords (B) for one female participant (F. M.). The *y*-axis indicates relative changes in concentration (from −1 × 10^−06^ to 5 × 10^−06^ mol/L) for HbO (thin line), HbR (dashed line), and HbT (thick line). Rows correspond to three different cerebral regions (frontal, temporal, and occipital region bilaterally). The NIRS channels in each region are indicated on the head templates. In each region, hemodynamic responses were averaged across the 13 blocks and across all the channels included in the region. The red line delineates the time interval during which the participants read the irregular words and nonwords (reading starts at time 0 and stops at 20 sec).

A typical hemodynamic response (HbO, HbR, and HbT concentrations) obtained with participant F. M. in reading aloud is illustrated in Figure 3A for irregular words and Figure 3B for nonwords for six different cerebral regions (frontal, temporal, and occipital regions bilaterally).

For this participant (F. M.), [HbO] and [HbT] values increased immediately after initiating the reading task, and gradually reached their peak at about 6 sec in the occipital regions bilaterally. In the frontotemporal regions bilaterally, [HbO] and [HbT] reached their peak concentrations at around 17 sec. When the participant stopped reading, [HbO] and [HbT] levels showed a rapid decline and gradually returned to their baseline. This pattern of increase was sustained for [HbR] albeit with a much smaller amplitude than [HbO]. We also found that [HbO] concentrations were higher in the left than the right hemisphere in the frontotemporal regions. Hemodynamic changes were analogous for irregular word and nonword reading. All participants showed similar patterns of hemodynamic responses except for B. B., who showed the reverse pattern. In this participant, we recorded a decrease in [HbO] and an increase in [HbR] concentrations in all regions but the bilateral occipital regions. We observed in another participant (J. T.) a right lateralization of reading in the frontotemporal regions with higher [HbO] concentrations in the right than the left hemisphere, for both irregular words and nonwords.

In irregular word reading, we found that the maximum peak of activation for [HbO] in all 12 participants occurred at about 8 sec in the bilateral occipital cortices, at about 12 sec in the bilateral temporal regions, and finally at about 14.5 sec in the bilateral frontal regions. In nonword reading, the maximum peak of activation we observed for the 12 participants occurred also at approximately 8 sec in the bilateral occipital cortices, but at about 13 sec in the bilateral frontal regions and approximately 14 sec in the bilateral temporal regions, in contrast with irregular word reading.

#### Spatial distribution of the significant hemodynamic responses

We performed a two-tailed paired *t*-test for each participant (*t*(12) > 2.17, *P* < 0.05, uncorrected) to determine the cerebral regions in which [HbT] concentrations in reading tasks were significantly different from those measured at rest (10-sec duration before the beginning of the reading tasks). [HbT] was chosen as the dependent variable because it correlates with cerebral blood flow. The regions were fitted on a segmented Brodmann Atlas template. The following regions were delimited for all participants: (1) the left prefrontal gyrus (BA46), (2) the IFG (BA44, 45), (3) the premotor and motor cortex (BA4, 6), (4) the somatosensory association cortex (BA5, 7), (5) the middle and superior temporal gyri (BA21, 22), (6) the angular gyrus (BA39), (7) the supramarginal gyrus (BA40), (8) the fusiform gyrus (BA37), and (9) the visual cortex (BA17, 18, 19), as well as their right homologous regions, for a total of 18 regions. We report individual results for the maximal *T*-value obtained in the 18 regions for irregular word and nonword reading in Table 2.

**Table 2:** Individual highest *T*-values measured in 18 cerebral regions (ordered from anterior to posterior) when comparing HbT concentrations in (A) irregular word reading versus rest (cross fixation) and (B) nonword reading versus rest (cross fixation)

Cerebral region		F.M.	I.C.	J.N.	J.T.	C.T.	J.P.	A.C.	D.V.	N.H.	B.B.	H.C.	M.D.
Left prefrontal cortex	A	5.63**				6.36***					5.39**		
	B	6.53***				6.63***					11.17***		
Right prefrontal cortex	A	5.47**				5.95***					3.73**		
	B	11.23***				8.70***					11.65***		
Left inferior frontal gyrus	A	16.73***	8.45***	4.12**	4.37**	5.46**	3.27**	4.83**	9.17***	2.68*	14.22***	2.88*	4.10**
	B	14.12***	8.6***	4.15**	4.40**	3.05*	4.61**	2.35*	11.06***	3.77**	15.23***	2.19*	4.04**
Right inferior frontal gyrus	A	10.69***	8.56***	9.18***	5.90***	2.37*	4.13**	4.70**	7.63***	6.04***	10.64***	2.56*	8.03***
	B	13.33***	5.83***	17.28***	4.96**	8.05***	4.17**	2.82*	10.19***	5.27**	13.62***	2.17*	11.19***
Left premotor and motor cortex	A	8.91***			5.11**			4.83**		6.05***	8.26***	7.58***	8.75***
	B	7.58***			4.92**			4.08**		6.26***	8.22***	8.61***	6.08***
Right premotor and motor cortex	A	6.11***			5.11**			5.99***		5.30**	6.85***	8.70***	8.21***
	B	5.93***			4.92**			3.31**		5.36**	11.47***	6.34***	9.29***
Left somatosensory association cortex	A		7.18***			2.7*		3.87**		5.14**	5.63**	7.31***	8.76***
	B		8.83***			4.47**		6.61***		4.79**	6.4***	7.51***	6.93***
Right somatosensory association cortex	A		8.13***			5.61**		3.19**		6.80***	6.92***	8.19***	6.68***
	B		10.03***			6.08***		7.15***		6.29***	4.45**	9.59***	8.75***
Left middle and superior temporal gyrus	A	16.72***	7.05***	11.82***	3.74**	2.81*	3.32**	7.06***	8.41***	4.93**	5.94***	5.24**	5.41**
	B	21.32***	4.42**	8.68***	3.24**	2.30*	2.68*	3.19**	9.20***	3.58**	4.43**	5.11**	4.29**
Right middle and superior temporal gyrus	A	13.5***	6.22***	10.01***	6.36***	2.85*	3.21**	9.64***	5.05**	4.67**	4.37**	3.36**	4.98**
	B	9.26***	4.14**	9.87***	3.35**	4.07**	2.7*	4.02**	8.47***	5.20**	7.5***	3.26**	4.82**
Left supramarginal gyrus	A							3.81**		3.95**		6.54***	7.89***
	B							4.65**		4.45**		5.41**	8.37***
Right supramarginal gyrus	A							2.72*		4.85**		8.76***	4.05**
	B							3.72**		5.22**		8.29***	5.01**
Left angular gyrus	A			5.82***		3.7**							
	B			5.33**		2.72*							
Right angular gyrus	A			5.71***		3.53**							
	B			5.60**		4.19**							
Left fusiform gyrus	A	3.07**		10.85***			4.46**		6.58***				
	B	2.18*		5.90***			5.84***		5.11**				
Right fusiform gyrus	A	5.03**		10.22***			4.48**		7.67***				
	B	5.25**		10.10***			4.20**		10.64***				
Left visual cortex	A	15.55***	10.26***	14.73***	9.54***	9.16***	5.78***	7.44***	4.59**	8.12***	12.21***	10.05***	12.96***
	B	14.28***	6.21***	9.10***	6.92***	12.51***	6.12***	8.07***	7.48***	6.17***	8.02***	6.36***	7.06***
Right visual cortex	A	7.55***	10.91***	12.02***	8.57***	7.63***	7.97***	3.73**	4.99**	7.93***	14.84***	8.62***	13.21***
	B	8.69***	12.44***	8.46***	8.86***	7.94***	5.02**	6.26***	8.28***	6.90***	11.38***	9.72***	11.21***

* *P* < 0.05;

** *P* < 0.01;

*** *P* < 0.0001.

**Figure 4:**
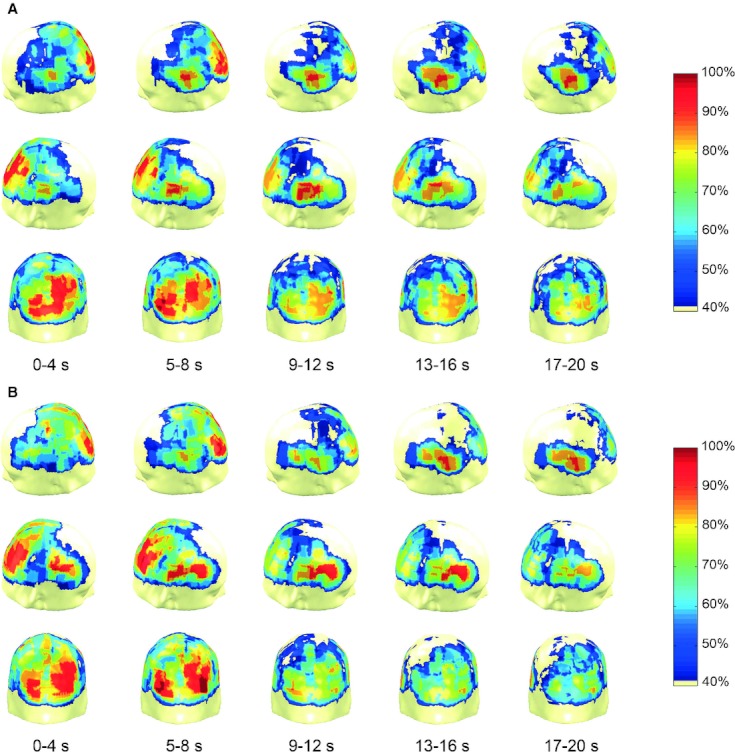
Mapping of the percentage of participants who had significant [HbT] *T*-value during reading aloud of irregular words versus rest (A) and during reading aloud of nonwords versus rest (B) as a function of regions and time intervals. The color scale goes from 40% (blue color) to 100% (red color) of the participants. The maps are projected on the skin for illustrative purposes. Rows correspond to three different views of the head (left, right, and back view). Columns correspond to 4-sec time intervals in the 20-sec reading block.

For each region, we calculated the percentage of the 12 participants who showed a significant *T*-value for [HbT] within 4-sec intervals. We mapped this percentage, starting at 40% (5/12 participants) as a function of region and time intervals in irregular word (Fig. 4A) and nonword (Fig. 4B) reading.

We found that over the total 20-sec irregular word and nonword reading task duration, all participants had significantly higher [HbT] concentrations than those measured at rest, in the bilateral inferior frontal gyri, middle and superior temporal gyri, and visual cortices (Fig. 4A and B). Between 40% and 70% of the participants showed [HbT] concentrations significantly higher or lower than that measured at rest in the bilateral prefrontal gyri, premotor and motor cortices, and somatosensory cortices. Interestingly, we observed that regions in which all participants showed significant *T*-values varied over both time intervals and stimulus type. In irregular word reading (Fig. 4A), participants showed significant *T*-values in the visual cortex bilaterally between 0 and 8 sec, in the right frontotemporal regions between 9 and 12 sec, and finally in the left frontotemporal regions between 13 and 20 sec. In nonword reading (Fig. 4B), we found that participants showed significant *T*-values in the visual cortex bilaterally between 0 and 4 sec, then in the right frontotemporal regions between 5 and 12 sec, and finally in the left frontotemporal regions between 13 and 20 sec. When comparing irregular words versus nonwords, more participants showed significant *T*-values in the left frontotemporal regions in irregular word than in nonword reading. In contrast, the reverse pattern of activation was observed in the right frontotemporal regions for which the percentage of participants was higher in nonword than in irregular word reading.

#### Effect of stimulus type on the spatial distribution of the hemodynamic responses

**Figure 5:**
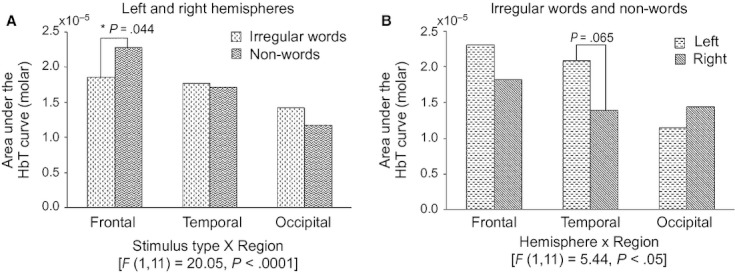
Significant stimulus type by region interaction (A) and hemisphere by region interaction (B).

We examined the effects of the stimulus type (irregular words vs. nonwords) as a function of laterality (left vs. right hemisphere) and the six regions that were commonly activated in 100% of the participants, that is: (a) the left IFG, (b) the left middle and superior temporal gyrus, and (c) the left visual cortex (with their right homologous regions). We ran an analysis of variance (ANOVA) using the area under the HbT curve as the dependent variable with three within-factors, Stimulus Type (irregular words and nonwords), Hemisphere (left and right), and Region (frontal, temporal, and occipital). We chose the area under the curve as it reflects the variations in the hemodynamic response in terms of both increases and decreases of [HbT] concentrations all along the 20-sec reading blocks. Statistical analyses were carried out on the SPSS statistics program, version 17.0 (SPSS Inc., Chicago, IL). The results revealed no significant effects of Stimulus Type (*F*(1,11) < 1), Hemisphere (*F*(1, 11) < 1) nor Region (*F*(1, 11) < 1) nor significant triple Stimulus Type × Region × Hemisphere interaction (*F*(1, 11) < 1). We did find significant double Stimulus Type × Region (*F*(1, 11) = 20.05, *P* < 0.0001) and Hemisphere × Region (*F*(1, 11) = 5.44, *P* = 0.025) interactions. The double interactions were further decomposed using post-hoc analyses to assess Stimulus Type and Hemisphere effects in each of the frontal, temporal, and occipital regions. For the Stimulus Type effect, we found higher [HbT] values in nonword than in irregular word reading in the frontal regions (*F*(1, 11) = 5.16, *P* = 0.044), whereas the differences in the temporal (*F*(1, 11) < 1) and in the occipital regions (*F*(1, 11) = 3.61, *P* = 0.084) were not significant. As for the Hemisphere effect, we observed a trend in the temporal region (*F*(1, 11) = 4.20, *P* = 0.065), with higher [HbT] values in the left than in the right hemisphere, but no significant differences in the frontal (*F*(1, 11) < 1) nor in the occipital regions (*F*(1, 11) = 2.250, *P* = 0.162) were found. Figure 5 illustrates the significant Stimulus Type by Region interaction (Fig. 5A) and Hemisphere by Region interaction (Fig. 5B).

We estimated that the Stimulus Type effect found in the frontal region, with higher [HbT] values in nonword than in irregular word reading, could be related to task performance. As reported in Table 1, all participants had a slower reading speed for nonwords than irregular words and also produced more errors in reading nonwords than irregular words. In an fMRI study, Mechelli et al. (2000) reported a strong positive linear effect of stimulus presentation rate (i.e., brain activity increased with presentation rate) during silent reading of words and pseudowords in the visual areas, the right superior temporal gyrus, and the bilateral precentral gyri. To assess the possibility that reading speed and error rate may influence hemodynamic responses in our study, we ran correlation analyses between [HbT] values recorded in bilateral inferior frontal gyri and reading speed, as well as correlation analyses between [HbT] values and error rate. Using Pearson's correlation, we found no significant correlation between the irregular word reading speed (mean number of stimuli read in 13 blocks) and the [HbT] values measured in the left (*r* = 0.136, *P* = 0.673) and right IFG (*r* = 0.008, *P* = 0.981), nor between the error rate (mean number of errors produced in 13 blocks) and the [HbT] values in the left (*r* = 0.314, *P* = 0.320) and right IFG (*r* = 0.030, *P* = 0.927). Similar results were obtained in nonword reading, with no significant correlation between the mean number of nonwords read and the [HbT] values in left (*r* = −0.075, *P* = 0.337) and right IFG (*r* = −0.304, *P* = 0.337), nor between the mean number of errors produced and the [HbT] values measured in the left (*r* = −0.049, *P* = 0.879) and right IFG (*r* = −0.076, *P* = 0.814).

## Discussion

The aim of this study was to investigate the applicability of an fNIRS protocol in studying the patterns of activation for the lexical and phonological pathways of reading. We chose the fNIRS technique because it is resistant to movement artifacts allowing the use of an overt reading task. We used irregular word and nonword stimuli because the former are most likely to activate the lexical pathway, whereas the latter can be read only through the phonological pathway. The results for [HbT] concentrations, measured in the total 0- to 20-sec time interval, revealed a significantly higher activation in the bilateral frontal regions in nonword than in irregular word reading. This was not correlated with the reading speed nor accuracy of the participants and is consistent with the fMRI study by Joubert et al. (2004) who reported higher activation in the bilateral frontal regions in silent reading of nonwords and low-frequency words compared with high-frequency words. However, our findings contrast with the results of an fMRI study conducted by Mechelli et al. (2005) who reported a left lateralized rather than bilateral difference between pseudowords and irregular words in a silent reading task. As explained in the Introduction, there were no fNIRS studies that compared the overt reading of irregular words and nonwords. In two fNIRS studies, the researchers tested participants in a lexical decision task involving the silent reading of words and nonwords (Kahlaoui et al. 2007; Hofmann et al. 2008). While Kahlaoui et al. (2007) found an increase in bilateral activation for nonwords in comparison with words, which included frontal and temporal regions in young adults and elderly participants, Hofmann et al. (2008), who only recorded hemodynamic responses in the left hemisphere, reported higher activation in the SFG and the IPG for words in comparison with pseudowords. In both of these fNIRS studies, the hemodynamic response reflected the whole processing from the silent reading of the stimuli up to the decision-making process. Because word stimuli can be recognized at the early visual orthographic stage, the participants must not access the sound form of words. Thus, it is conceivable that the reported differences between words and nonwords in these studies reflect the activation of the sound form for nonwords but not for words. Similarly, as the researchers could not verify whether the participants accessed the sound form in both types of stimuli, the differences reported in fMRI studies between the covert reading of word and nonword stimuli should be questioned. To address these caveats, in our experimental paradigm, participants had to read both words and nonwords out loud, allowing us to measure their reading performance. Moreover, word and nonword stimuli were strictly matched to visual characteristics (number of letters) and phonological complexity (number of phonemes, number of syllables, and syllabic structures). In doing so, we ensure that the visual and phonological features susceptible to interfere with the reading process are controlled, both at the early visual and at the late articulatory output stages. This allows us to record the hemodynamic responses that are specifically associated with the lexical and phonological pathways of reading, under the best conditions. We attribute the difference in activation found in the bilateral frontal regions, which was higher for nonwords than for irregular words, to the grapheme-to-phoneme conversion associated with the phonological pathway of reading. Nonetheless, because the overt reading of irregular words and nonwords stimuli may differ from the early visual processing stage up to the articulatory output, the hemodynamic responses that were recorded in a 20-sec time interval reflected the whole processing, including the grapheme-to-phoneme processing. Further investigation is required before we may draw firm conclusions about the localization of the brain regions that were involved in the grapheme-to-phoneme conversion.

An advantage of the fNIRS over the fMRI technique is that the temporal course of the activation can be examined. Using this, we analyzed the activation across five time intervals in the right and left visual, temporal, and frontal regions. The results indicated that most participants showed significant bilateral activation in the visual cortex for irregular words and nonwords in the early time interval (0–8 sec), moving to the right frontotemporal regions in the intermediate time interval (9–12 sec). In the late time interval (13–16 sec), we observed more participants with significant activation in the left IFG for irregular words and in the right IFG for nonwords. This latter hemispheric difference was lost when we averaged the hemodynamic responses over the entire (0–20 sec) time interval with the ANOVA revealing higher HbT values in frontal regions in nonword reading than in irregular word reading, regardless of the hemisphere. Our interpretation for this apparent contradiction between static and dynamic analyses is that in the dynamic analysis, the lateralization was estimated by the number of participants who showed HbT values significantly different from baseline, whereas in the static analysis, the dependant variable in the ANOVA was the area under the HbT curve value for each participant. Individual differences in language hemispheric dominance may also have contributed in masking the hemispheric difference in the frontal regions. For instance, as reported above, one right-handed participant (J. T.) has a right-hemisphere dominance for language, which was confirmed by an fMRI procedure.

Another advantage of the fNIRS is that both [HbO] and [HbR] concentrations can be measured, whereas the fMRI is limited to measuring only [HbR] concentrations. Typically, in healthy participants, regional cerebral blood flow (rCBF) is increased by the neural activity resulting in an increase in [HbO] and [HbT] concentrations, with a decrease in [HbR] concentrations (Sakatani et al. 1998). A number of fNIRS studies, however, documented other patterns in the relative concentration of HbO and HbR during verbal tasks. Watanabe et al. (1998) recorded an increase in [HbR] in the inferior frontal lobe when participants were tested in a written verbal fluency task and Yamamoto and Kato (2002) reported increases in [HbO], [HbR], and [HbT] in the Broca area in a word repetition task. Lo et al. (2009) observed a significant [HbO] increase in an overt text reading task with no variation in the [HbR] concentration. In our experiment, most of the participants showed a typical increase in [HbO] and [HbT] when they began to read, with a return to the baseline level once they stopped reading, and this in all cerebral regions known to be involved in reading. Three of the participants showed a reverse pattern of activation, that is, a decrease in [HbO] with an increase in [HbR] in the bilateral prefrontal and frontotemporal regions (B. B.), and in the bilateral prefrontal gyri (F. M. and C. T.). Similar patterns of hypooxygenation were reported in Liu et al. (2008)'s study when participants read out loud a nonfamiliar text. Six of the 22 participants showed hypooxygenation in the left prefrontal region and three in the right one. In accordance with Liu et al. (2008), three scenarios could account for the recording of hypooxygenation: (a) the vascular steal mechanism (e.g., Sakatani et al. 1998), (b) the possibility that we detected the activity in an area adjacent to the activated region, or (c) the hypooxygenation represented a deactivation of the cortical area (e.g., Hoshi et al. 1994; Sakatani et al. 1998). However, a better understanding of the neurophysiological mechanisms during neuronal activity is needed before we can evaluate the relative contributions of each of these scenarios in the hypooxygenation phenomenon.

To conclude, our findings indicate that the fNIRS technique is a suitable tool for the examination of performance in overt reading. The advantage of this reading task is that it can be adapted to the participant level. The rate of presentation of the stimuli can be adjusted to the individual reading speed and the list of stimuli to the reading level of the participants, for assessment of young children with developmental dyslexia and adults with developmental or acquired dyslexia for instance. This language task is also ideal to assess the effect of speech therapy or transcranial magnetic stimulation (TMS) treatment as one can easily create an equivalent list of stimuli to avoid facilitatory effects in test–retest. The overt reading task will be added to the protocol for the presurgical examination of epileptic patients in our laboratory. The present protocol includes tasks for the assessment of the expressive and receptive oral language, but no tasks for the assessment of the written language. Further investigation will include the testing of patients with primary reading epilepsy.

## Supporting Information

**Appendix.** List of irregular words (A) and nonwords (B) in the 13 blocks.
