# Supplementary material for: Functional near-infrared spectroscopy for the assessment of overt reading
Source: Brain Behav. 2012 Oct 28;2(6):825–37. doi: 10.1002/brb3.100 (PMC3500469; doi:10.1002/brb3.100)
Supplement: Supplementary file 1 [file brb30002-0825-SD1.doc]

1	A	chef, perdrix, pachyderme, accord, indolent, septembre, pentagone, dolmen, archéologue, coyote, tennis, gnôle, amen, parasite, gens, baptême, sosie, œsophage, correct, lascif, gros, qualité, pistil, gemme, obus, prix, emmener, coller, magenta, pays	
	B	jist, nomus, adoïne, quintot, olitié, unquille, voairrier, paccyx, sartofidat, hazer, fagande, squane, goyen, oguépique, drot, cripos, nillian, pasonis, arpum, scartel, bros, pimoë, biremme, corc, ouduille, kref, quoas, nenher, ipoudus, brinat	
2	A	gars, geôlier, almanach, œillet, infarctus, grésil, coopté, laser, anorexie, matelas, calcium, lier, inuit, archevêque, chiasme, héros, tabac, pagayer, nombril, amas, toast, baptiser, hennir, schisme, jadis, stop, chahut, lascar, orchidée, rébus	
	B	dric, exquos, alquaïche, dageule, tondabat, oyie, aigolerre, opplit, tsychalovie, opis, ansprit, gred, fospens, dondani, quol, huyan, dolium, imimis, hanni, uther, gaurg, biagnestec, ochan, prop, hamos, vonc, rubsisde, ercool, cholagnerd, harnos	
3	A	champ, brebis, quotidien, banjo, requiem, soya, immangeable, direct, machiavélique, cadenas, pitié, presse, galop, équité, juin, rebours, podium, examen, monsieur, pupille, croc, salsifis, milliard, ville, chorale, hier, cognac, gentil, oxygène, cassis	
	B	scef, torbrix, tochyterne, éttart, anbilent, feptondre, pendagine, balmen, arquaérique, toyite, pennos, gnêre, onen, tirasate, jans, diptène, sisue, oevaphige, tallect, riscef, dris, quarito, tospil, zemme, ubos, drix, ommenet, pirrer, nojenpa, fays	
4	A	Bloc, exquis, archaïque, gageure, candidat, oyez, aquarelle, accroc, psychologie, abus, inscrit, bled, suspens, bandana, quiz, hayon, radium, ananas, henné, éther, bourg, diagnostic, agent, trop, humus, jonc, subsiste, alcool, charognard, hormis	
	B	tars, geôriet, olnimach, oeillat, onserctus, drasil, toospé, joser, onalexie, motelis, tirsium, vier, amuit, olchefêque, quiosme, horis, padac, tapayer, mandril, inis, poast, doptaser, hannor, schasme, vodis, stip, fouhat, riscar, alchibou, lédus	
5	A	drap, vingt-deux, chrysanthème, mazout, prospectus, ennemi, estomac, pathos, coïncidence, sculpture, tapis, mille, ancien, oasis, tronc, whisky, chef-d'œuvre, délicat, païen, samba, bille, abdomen, croquis, sang, chariot, bras, boycott, habit, processus, pizza	
	B	guos, doyou, paoutchonc, fimus, ibaguit, dêchos, eugalium, guirum, toechifiant, obtis, grajet, saon, samptieux, albotris, gname, magnot, vaison, pithater, thirpus, pictas, voug, sitiéta, pronquille, gneu, rochen, piors, titoye, natis, térasil, doseps	
6	A	choc, forum, abbaye, villa, ischémie, sérum, pharaon, client, aérosol, talus, œdème, sept, atlas, œstrogène, femme, dompteur, quota, immanence, poncho, campus, rhum, séquoia, accent, crypte, exemple, fret, escroc, pastis, aiguiser, scorbut	
	B	plos, lospect, iquotour, ropus, chonquilla, psachogue, jarendiers, wadin, parritilum, toril, ferpras, prerc, pormit, onafrer, rips, tancept, padens, ranguistèque, feumil, pamptour, squiw, poptasont, hiapos, heut, alchit, droc, oubit, garil, siplomen, chissos	
Appendix. List of irregular words (A) and non-words (B) in the 13 blocks.
7	A	chas, boyau, caoutchouc, sinus, adéquat, gâchis, aquarium, quorum, coefficient, obtus, brevet, faon, somptueux, albatros, gnome, magnat, faisan, cathéter, thermos, cactus, joug, satiété, tranquille, gnou, lichen, tiers, papaye, métis, parasol, biceps	
	B	grap, songt-soux, chroventhane, novout, tralpicpus, emmina, aspémac, thapos, toïnfitince, scolptère, potis, nille, ontien, aésis, pronc, whospi, guet-d'oiffre, bilétot, taïen, fambo, dille, iptanin, traquis, fong, failiet, glis, goypott, hibot, tracerrus, tozzi	
8	A	clos, respect, équateur, lotus, chinchilla, psychique, volontiers, wagon, curriculum, péril, surplus, clerc, carnet, enivrer, laps, concept, dépens, linguistique, chenil, compteur, squaw, captivant, hiatus, août, archer, troc, audit, baril, cyclamen, châssis	
	B	guoc, solum, oddohi, gilla, aschénée, firum, phélaon, flient, aoléfir, pilus, oebime, fept, otlis, oestravile, semme, bomptour, guipo, ommanince, tanchi, pombis, phum, féquoio, opsint, grapte, exompre, gret, asproc, tostis, ouguiver, scaldit	
9	A	quille, corpus, ecchymose, avis, condamner, septième, coréen, exil, asymétrie, loyer, charisme, test, fusil, occiput, mars, sceptique, toujours, millénium, enfer, fécond, isthme, pardessus, faucon, sceptre, lilas, thym, suspect, étang, architecte, sourcil	
	B	gouc, dirit, tapahuire, waourp, guilaré, puster, aroxir, éfril, ilanonium, opors, totille, stind, himoc, toripis, baon, foixande, eupamne, velorter, tergis, tallen, mier, schoviprhane, tansil, mong, noyen, faoul, olos, quandet, goleyer, fauris	
10	A	moelle, gentiane, placebo, choyer, exotique, village, catéchisme, sangsue, cholestérol, écho, persil, cerf, igloo, indécis, donc, début, parfum, citoyen, quatuor, soldat, nerf, bégayer, gymnase, raid, gadget, paye, ennui, orchestre, archétype, radis	
	B	tied, omper, tilodis, pihuer, ovendi, veûmer, orpisect, mempa, rhomiférus, fobtil, rynchée, eurs, nicho, paurlefis, pher, sosceste, vundo, nyovodis, toiller, panfus, dref, astarier, atium, naid, sconber, usthme, schamu, nébium, ibagio, teuvet	
11	A	bouc, délit, cacahuète, yaourt, choléra, permis, élixir, avril, aluminium, épars, papille, stand, hamac, pilotis, paon, soixante, automne, revolver, parvis, pollen, nier, schizophrène, cancer, gang, moyen, saoul, iris, quintet, balayer, souris	
	B	chille, parcus, acchynase, ojas, tindomner, foptièle, daléen, ixal, éfynétrie, royer, phirasme, pest, sovil, accotut, nirs, sciptogue, peuvours, nillanium, onter, sacand, osthme, torbessus, soucon, sciptre, raris, phym, fistect, oteng, irchapacte, feurcil	
12	A	pied, imper, paradis, cahier, agenda, jeûner, artéfact, mambo, rhinocéros, subtil, lyncher, ours, macho, tournevis, cher, fasciste, jumbo, myosotis, cuiller, confus, bref, escalier, opium, laid, scinder, asthme, schéma, médium, adagio, cachet	
	B	noerre, vontière, cricedo, chuyer, exitoque, fillèse, taquéfasme, fangsie, quaresdéral, acho, torsil, verf, abloo, anbéfis, bonc, bédat, talpum, fapoyen, quapiar, forbat, merf, dibayer, vymnèse, vaid, dodget, taye, annio, archispre, orquipyte, rudas	
13	A	zest, minus, égoïne, quantum, initié, anguille, joaillier, coccyx, certificat, hiver, seconde, squale, doyen, aquatique, brut, propos, million, cosinus, album, scalpel, gris, canoë, dilemme, parc, aiguille, clef, chaos, menhir, autobus, granit	
	B	quomp, dredos, quapibien, danzo, lequiam, foya, ommouvample, bolect, nochiésalique, tidemis, tatié, cresse, dorap, oquita, vuin, lidours, tabium, axémon, nonfieur, turille, proc, folfasis, nillièrd, tille, charile, hial, tignoc, vontil, axyvine, pichos	
